# Longitudinal Myocardial Deformation Does Not Predict Single Ventricle Ejection Fraction Assessed by Cardiac Magnetic Resonance Imaging in Children with a Total Cavopulmonary Connection

**DOI:** 10.1007/s00246-017-1753-z

**Published:** 2017-10-25

**Authors:** L. P. Koopman, L. M. Geerdink, S. S. M. Bossers, N. Duppen, I. M. Kuipers, A. D. ten Harkel, G. van Iperen, G. Weijers, C. de Korte, W. A. Helbing, L. Kapusta

**Affiliations:** 1 Division of Pediatric Cardiology, Department of Pediatrics, Erasmus Medical Centre, Sophia Children’s Hospital, Rotterdam, The Netherlands; 2 Department of Pediatric Cardiology, Radboud University Medical Centre, Amalia Children’s Hospital, Nijmegen, The Netherlands; 3 Department of Pediatric Cardiology and Intensive Care Medicine, Hannover Medical School, Hannover, Germany; 4 Department of Pediatric Cardiology, Academic Medical Centre, Amsterdam, The Netherlands; 5 Department of Pediatric Cardiology, Leiden University Medical Centre, Leiden, The Netherlands; 6 Department of Pediatric Cardiology, University Medical Centre Utrecht, Wilhelmina Children’s Hospital, Utrecht, The Netherlands; 7 Department of Radiology, Erasmus Medical Centre, Rotterdam, The Netherlands; 8 Pediatric Cardiology Unit, Dana Dwek Children’s Hospital, Tel-Aviv Sourasky Medical Center, Affiliated to the Sackler Faculty of Medicine, Tel-Aviv University, Tel Aviv, Israel

**Keywords:** Fontan, Total cavopulmonary connection, Cardiac MRI, Speckle tracking echocardiography

## Abstract

Survival of children with single ventricle heart defects after the total cavopulmonary connection (TCPC) has improved, but impaired cardiac function remains a major cause of morbidity and mortality. Cardiac magnetic resonance imaging (cMRI) is the gold standard in assessing single ventricle volume and function, but high costs and limited availability hamper its routine use. A cheaper and more available alternative is echocardiography. Myocardial function can be studied in more detail using speckle tracking echocardiography (STE). The purpose of the study was to describe the association between myocardial deformation assessed by speckle tracking echocardiography (STE) and single ventricle function assessed by cMRI and to evaluate differences in myocardial deformation in children with single left and single right ventricular morphology. Cross-sectional, multicenter study in 77 children after TCPC was conducted. STE segmental and global longitudinal peak strain and systolic strain rate (SR) of the dominant ventricle were measured. Impaired SV function by cMRI was defined as ejection fraction (EF) < 45%. Mean age was 11.8 (range 9.7–14.3) years. Pearson R for cMRI EF versus global longitudinal strain and SR was − 0.25 (*p* = 0.06) and − 0.03 (*p* = 0.82), respectively. Global single ventricle longitudinal strain and SR was similar in patients after TCPC with single left and single right ventricular morphology (− 19.0 ± 3.1% vs 19.2 ± 3.2%, *p* = 0.94). STE myocardial deformation parameters do not correlate with single ventricle ejection fraction assessed by cMRI.

## Introduction

The Fontan circulation is a palliative surgical strategy for children with congenital heart disease who cannot be offered a biventricular repair [1]. The Fontan circulation in the current era is achieved using a staged approach by connecting the superior caval vein and inferior caval vein (either by an extra cardiac conduit [ECC] or intra-atrial lateral tunnel [ILT]) directly to the pulmonary arterial circulation, resulting in a total cavopulmonary connection (TCPC) [2].

While the short- and median-term survival in children palliated with a TCPC has improved considerably during the last decades [3], it is well recognized that cardiac function and exercise capacity in children with a TCPC is reduced as compared to healthy children and may deteriorate over time [4].

Cardiac magnetic resonance imaging (cMRI) is considered to be the gold standard for the non-invasive assessment of single ventricle volume and function, although its routine use is hampered by high costs, long analysis time, lack of availability, need for sedation in small children, and inability to use in most patients with pacemaker devices [5]. Echocardiography is cheaper, faster, widely available, and can be used in non-sedated children and patients with pacemaker devices. However, standard 2-dimensional echocardiographic measurements of ventricular volume and function such as ejection fraction (EF) by biplane Simpson and M-mode shortening fraction cannot be used in the single ventricle due to its different geometry as compared to the normal left ventricle (LV). Speckle tracking echocardiography (STE)-derived myocardial deformation [strain and strain rate (SR)] is a less geometry-dependent technique to assess myocardial function compared to standard echocardiographic methods [6]. Various, yet rather small studies in children with a TCPC, have been published, suggesting that measurement of myocardial deformation is feasible and reproducible, and that lower strain and SR values are measured in patients versus controls as well as in TCPC patients with single right ventricular (RV) versus single LV morphology [7–9]. It is unknown whether myocardial strain and SR parameters provide additional information on single ventricular function in patients with a TCPC assessed by cMRI.

The purpose of this study is to describe the association between longitudinal STE-derived myocardial deformation parameters and single ventricle function assessed by cMRI in a relatively large cohort of contemporary pediatric TCPC patients and to evaluate differences in myocardial deformation in patients with single right versus single left ventricular morphology.

## Methods

### Patients

We performed a cross-sectional multicenter study in single ventricle patients with a TCPC. Details of the recruitment procedures and in- and exclusion criteria are described elsewhere [10]. In short, a total of 77 patients after the TCPC procedure were included. Inclusion criteria were as follows: having undergone a TCPC through a staged approach with a partial cavopulmonary connection procedure prior to the completion of the TCPC according to a current technique (ILT or ECC); completion of the TCPC before the age of 7 years and inclusion in the study at an age of at least 8 years or older; and a minimum of 3 years of follow-up since completion of the TCPC. Patients with mental retardation were excluded from this study. Patients were recruited from four tertiary referral centers in the Netherlands that used the same ultrasound equipment (center that used another ultrasound system was excluded). The study was approved by the institutional medical ethical review boards of the participating centers. Written informed consent was obtained from all patients and/or their parents. All patients underwent routine physical examination, including body weight, height, blood pressure.

### Echocardiographic Assessment

All patients underwent a detailed transthoracic echocardiographic examination performed by an experienced technician following the recommendations of the American Society of Echocardiography [11]. All images were obtained with an appropriate transducer on locally available machines (GE Vivid7; GE Vingmed Ultrasound AS, Horten, Norway) according to a standardized protocol that was used in all participating centers. Images were reviewed and supervised by an experienced cardiologist (LK) in the core lab situated in one location.

#### Conventional Echocardiographic Measurements

Analyses were performed offline using EchoPAC version 11.0 (GE Healthcare, USA). All measurements were performed using 3 different cardiac cycles and the values were averaged. Pulsed wave Doppler flow measurements were obtained at the right upper pulmonary vein. Continuous wave Doppler flow velocities of the ascending (neo) aorta and descending aorta were obtained. Pulsed wave Tissue Doppler measurements were obtained at the basal segment of the lateral wall of the dominant ventricle. Annular plane systolic excursion (APSE) was assessed using M-mode interrogation of the lateral side of the dominant AV valve.

#### Speckle Tracking Echocardiography (STE) Image Acquisition and Off-Line Analysis

Apical grayscale images were acquired at frame rates of 55–90 frames/s. The images were analyzed by a single investigator (LMG) using EchoPAC (version 11.2, GE Vingmed Ultrasound, Horten, Norway) according to the vendor’s instructions and as previously described by our group [12]. In short, endomyocardial borders of the dominant ventricle were manually traced within the end-systolic frame. Secondly, epicardial tracing was automatically generated by the computer algorithm and, when necessary, manually adjusted to cover the whole myocardial wall. The tracking algorithm then followed the myocardial speckles during the cardiac cycle. Tracking was accepted only if both visual inspection as well as the software indicated adequate tracking and strain curves. The software automatically divided the apical chamber views in 6 segments (3 septal and 3 lateral). Strain curves of 3 separate cardiac cycles were averaged after equalizing cycle lengths, by padding zeros behind the diastolic phases of the shorter cycles [13]. To determine global longitudinal strain and SR, the average of the six segments was calculated and at least 4 out of 6 valid lateral segments were needed. To determine septal or lateral wall strain and strain rate, at least 2/3 segments should be included.

### Cardiac Magnetic Resonance Imaging (cMRI)

The participants underwent a cMRI study on the same day as the echocardiographic study. Patients with pacemaker devices were excluded for undergoing cMRI. The ventricular volumes were imaged using a multi-slice, multiphase, steady-state, free precession sequence as previously reported [10]. In short, the series were acquired at rest and volumes were analyzed using an Advanced Windows workstation (GE Medical Systems, Milwaukee, Wis) and MASS software (Medis Medical Imaging Systems, Leiden, The Netherlands). The endo- and epicardial contours were manually drawn in the end-diastolic and end-systolic phases. The volumes, EF, and mass of the LV and RV were added to calculate the single ventricular indices to compare the different cardiac configurations possible. Cardiac index (CI) was calculated by multiplying stroke volume with heart rate. Abnormal single ventricle EF was defined as < 45%.

### Reproducibility

Inter-observer and intra-observer variability of global longitudinal strain measurements were assessed in 10 randomly selected studies. To assess intra-observer variability, the same observer who performed all the measurements re-measured the same segments at least 2 weeks later to avoid recall bias (LMG). To assess inter-observer variability, deformation parameters were measured by one independent and blinded observer (LK). Reproducibility was expressed as bias (limits of agreement) and the coefficient of variation, COV (the standard deviation of the difference of paired samples divided by the average of the paired samples times 100).

### Statistical Analysis

Statistical analysis was performed using SPSS 21.0. (IBM corporation, New-York, USA). Normal distribution of the data was tested using Shapiro-Wilk test. Data are expressed as frequencies, mean (standard deviation) in case of normal distribution, or median (interquartile range) in case of non-normal distribution. Comparisons between groups were made using the independent *T* test (all comparisons were normally distributed). Categorical data are presented as counts and percentages. Pearson’s correlations and their statistical significance were calculated as measures of raw associations between measurements. Two-sided *p* values ≤ 0.05 were considered to be statistically significant.

## Results

### General Characteristics

Table 1 shows the general characteristics and conventional echocardiographic parameters of the whole study population. No significant Fontan baffle leakage was observed in our study population which was also represented by the high median oxygen saturation of 96%. Five participants had a epicardial pacemaker implanted due to heart block or sinus node dysfunction. Four children were diagnosed with right atrial isomerism and 1 child with left atrial isomerism. Two participants had a mildly increased flow velocity in the ascending aorta (1.6 and 2.1 m/s, respectively) and 3 participants had a mildly increased flow velocity in the descending aorta (1.9, 1.9, and 2.1 m/s, respectively). No patient had signs of clinically significant pulmonary vein stenosis at the time of assessment.

**Table 1:** General characteristics of the study population (*n* = 77)

Gender male	43 (55.8%)
Age at study (years)	11.8 (9.7–14.3)
Age at TCPC (years)	3.3 (2.5–3.9)
BSA (m^2^)	1.27 (1.09–1.53)
Resting HR (beats/min)	76 (68–88)
Resting SaO_2_ (%)	96 (94–97)
SBP (mm Hg)	112 (104–118)
DBP (mm Hg)	67 ± 9
Dominant LV/RV morphology	55/22
Single ventricle diagnosis	
HLHS	11 (14.3%)
RV hypoplasia with TA or PA	32 (41.6%)
DILV	8 (10.4%)
DORV. no TGA	6 (7.8%)
DORV. with TGA	2 (2.6%)
Other	18 (23.4%)
TCPC type (ECC/ILT)	11/66
Conventional echo parameters of dominant ventricle	
S′ velocity (cm/s)	5.9 (4.9–6.6)
APSE (mm)	11.5 ± 2.4

*APSE* annular plane systolic excursion, *BSA* body surface area, *DBP* diastolic blood pressure, *DILV* double inlet left ventricle, *DORV* Double outlet right ventricle, *ECC* extra cardiac conduit, *HLHS* hypoplastic left heart syndrome, *HR* heart rate, *ILT* intra-atrial lateral tunnel, *LV* left ventricle, *PA* pulmonary atresia, *RV* right ventricle, *S*′ systolic velocity, pulsed Tissue Doppler, *SaO*_*2*_ oxygen saturation, *SBP* systolic blood pressure, *TA* tricuspid atresia, *TCPC* total cavopulmonary connection, *TGA* transposition of great arteries

### Feasibility and Reproducibility

**Fig. 1:**
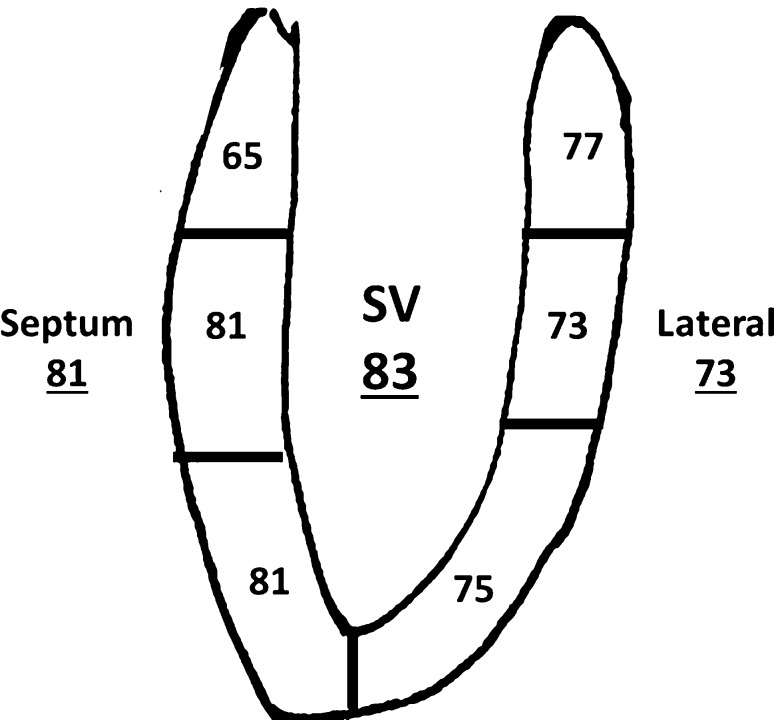
Feasibility of speckle tracking echocardiography in patients with a single ventricle. Numbers without underscore represent % of segments that could be measured. Numbers with underscore represent feasibility of measuring septum or lateral wall (at least 2/3 segments) and global measurement (at least 4/6 segments)

Feasibility of STE was highest for mid and apical septal segments and lowest for the basal septal segment (due to frequent absence of the basal septum in the setting of a large ventricular septal defect), Fig. 1. Reproducibility data for longitudinal myocardial deformation parameters of the lateral wall are provided in Table 2. COVs of myocardial deformation were higher when individual segments were analyzed compared to global parameters (data not shown).

**Table 2:** Intra- and inter-observer reproducibility for global myocardial deformation parameters

	Global longitudinal strain			Global longitudinal SR		
	Bias (%)	Limits of agreement (%)	COV (%)	Bias (1/s.)	Limits of agreement (1/s.)	COV (%)
Intra-observer	− 0.65	− 4.4 to 3.3	10.9	0.02	− 0.11 to 0.16	6.0
Inter-observer	0.63	− 3.5 to 4.8	12.0	− 0.03	− 0.32 to 0.27	13.2

*COV* coefficient of variation, *SR* strain rate

### The Association Between Myocardial Deformation, cMRI and Conventional Echo Parameters

**Fig. 2:**
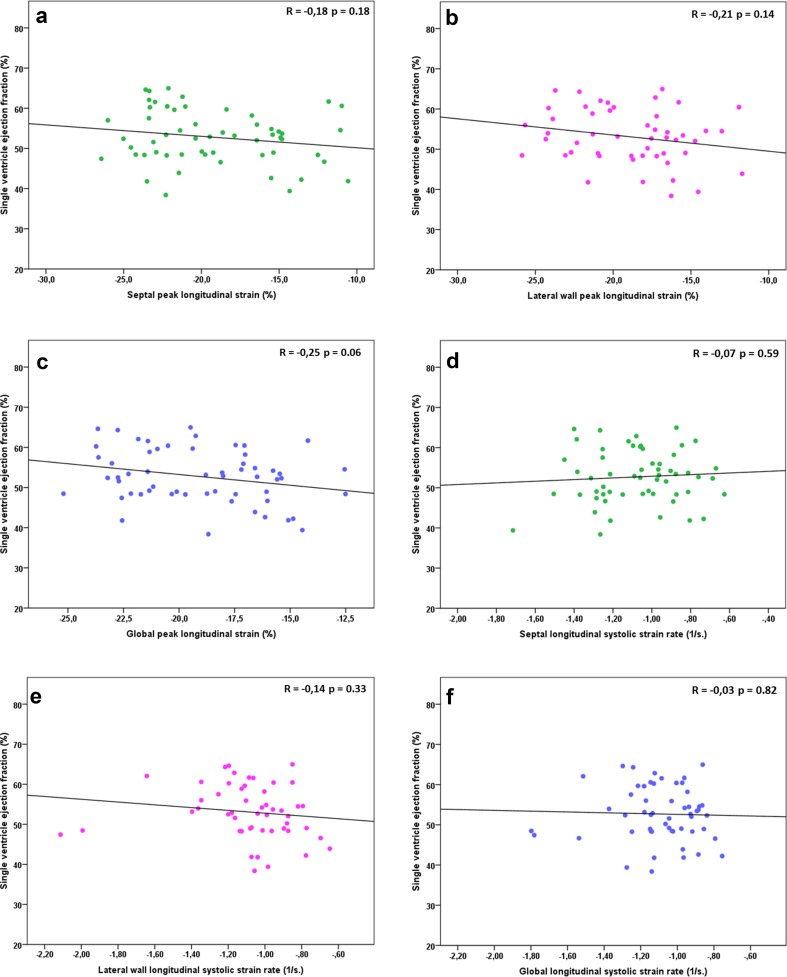
Association between single ventricle ejection fraction by cMRI and longitudinal peak strain of the septum (**a**), lateral wall (**b**), and global (**c**); longitudinal systolic strain rate of the septum (**d**), lateral wall (**e**), and global (**f**)

**Fig. 3:**
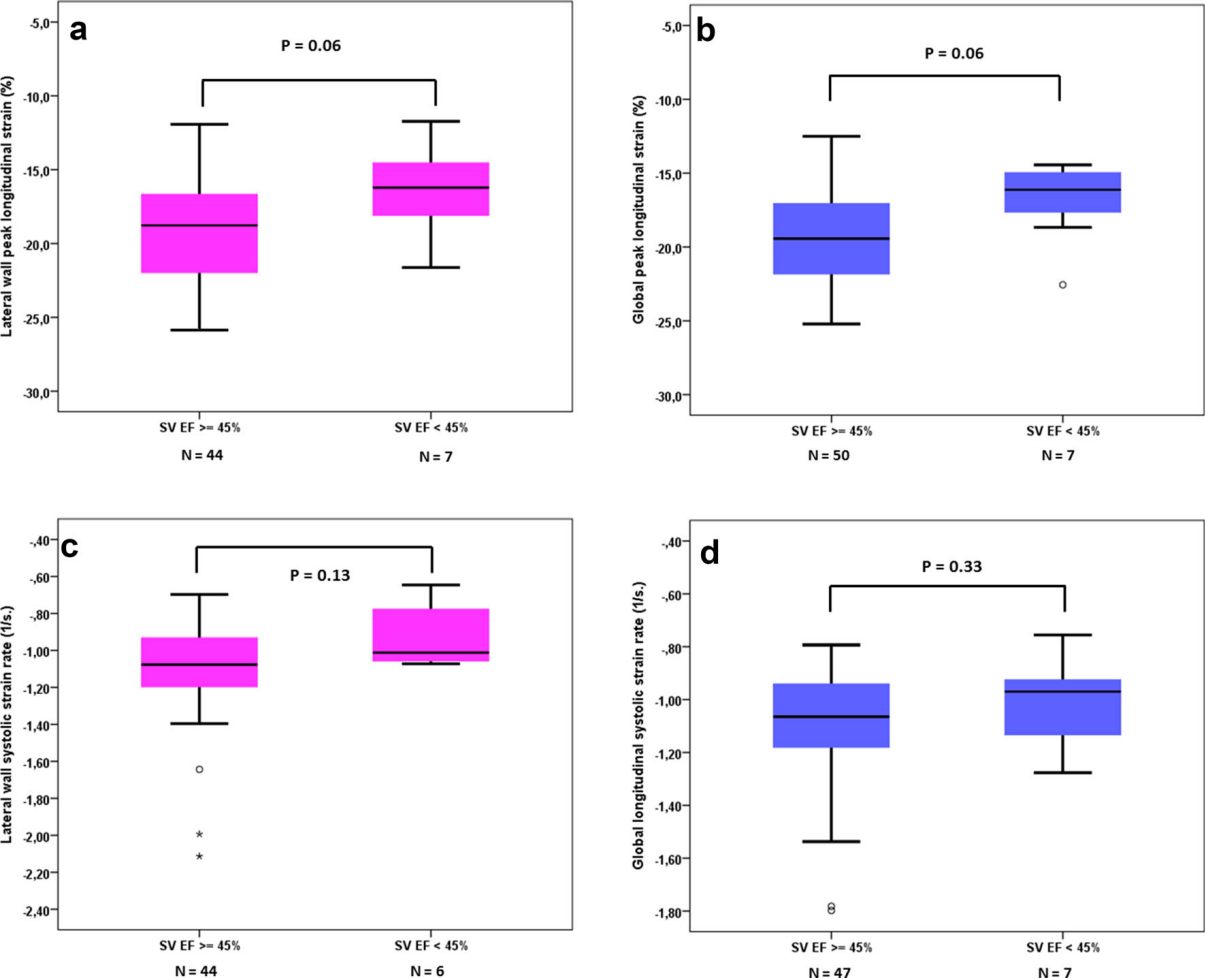
Lateral wall longitudinal strain (**a**) and strain rate (**c**) and global longitudinal strain (**b**) and strain rate (**d**) in children with a TCPC with normal EF (≥ 45%) and abnormal EF (< 45%) by cMRI

**Fig. 4:**
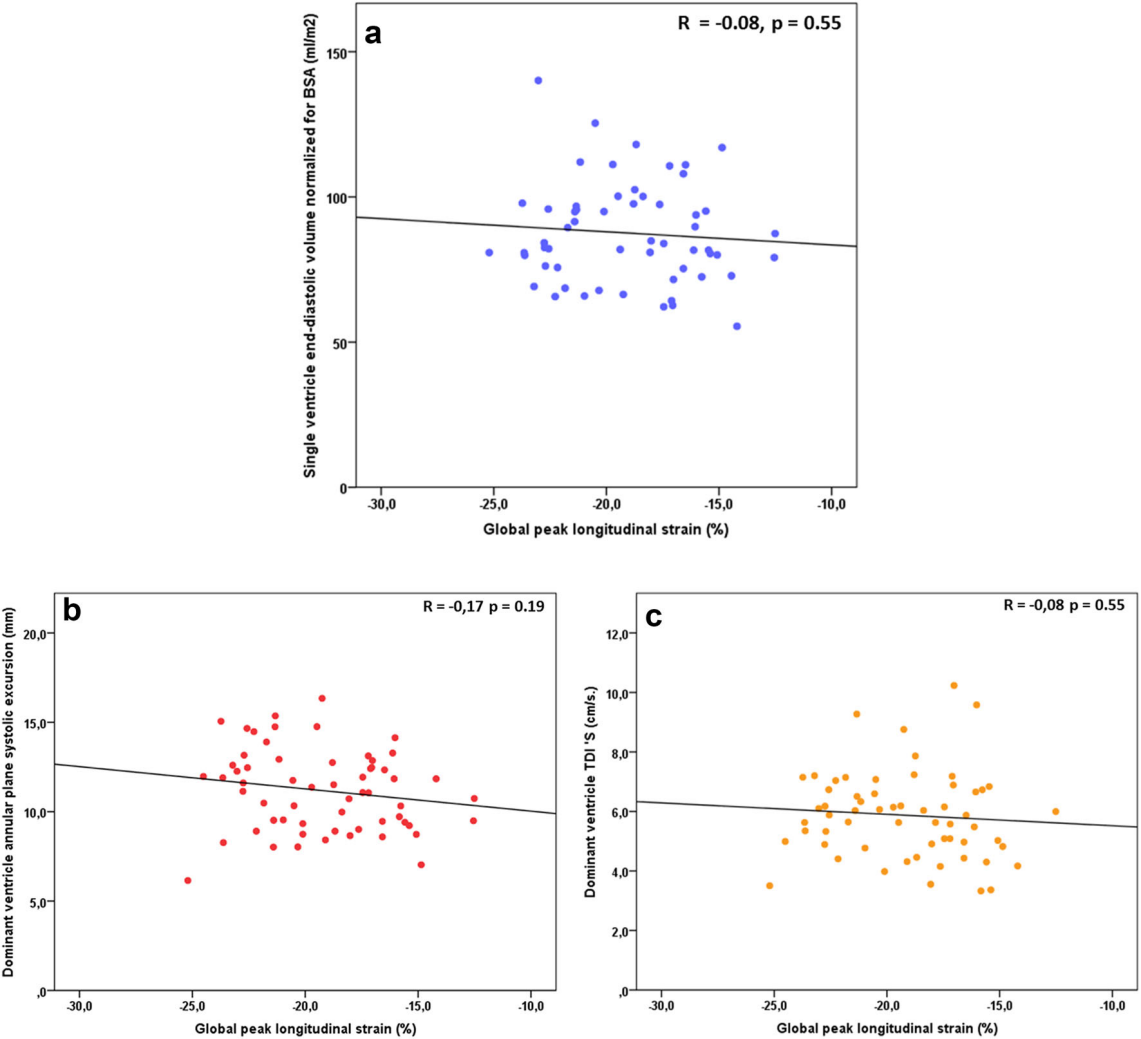
Correlation between global peak longitudinal strain and single ventricle end-diastolic volume normalized for BSA (**a**); dominant ventricle annular plane systolic excursion (**b**) and dominant ventricle systolic velocity, pulsed Tissue Doppler (S′) (**c**)

In Fig. 2, the associations between single ventricular function assessed by cMRI EF and longitudinal myocardial deformation parameters are shown. There was a trend towards lower (less negative) longitudinal strain values in children with lower EF, but none of the associations were statistically significant. No association was found between cMRI EF and STE strain rate (Fig. 1d–f). Figure 3 shows the results for lateral wall and global longitudinal strain and strain rate when children with a TCPC were dichotomized in a group with normal EF and abnormal EF by cMRI. Complete data on echocardiography and cMRI were available in 50 to 57 out of 77 participants and no statistically significant differences in myocardial deformation were found between children with normal and low single ventricle ejection fraction. Also, no association was found between global longitudinal strain by STE and cMRI SV volume (Fig. 4a), more conventional echocardiographic function parameters SV APSE (4B), SV S’ by pulsed wave TDI (4C) and other cMRI and conventional echocardiographic parameters (Table 3). Longitudinal systolic strain rate was unrelated to SV volumes by cMRI and echocardiographic parameters (Table 3). When septal or lateral wall longitudinal strain or strain rate was correlated to the parameters shown in Table 3 similar results were found (data not shown). A weak correlation was found between cMRI EF and SV APSE (Pearson R 0.34, *p* = 0.01). No association was found between cMRI EF and SV S’ (Pearson R 0.17, *p* = 0.19).

**Table 3:** Correlation between global longitudinal peak strain or strain rate and cMRI parameters and conventional echocardiographic parameters

	Global longitudinal strain		Global longitudinal strain rate	
	*R*	*P*	*R*	*P*
SV end-diastolic volume/m^2^	− 0.08	0.55	0.06	0.67
SV cardiac index	− 0.25	0.06	− 0.23	0.09
S′ velocity	− 0.08	0.55	− 0.22	0.11
APSE	− 0.17	0.19	− 0.10	0.47

*APSE* annular plane systolic excursion, *S*′ systolic velocity, pulsed Tissue Doppler

### Single Ventricle Morphology and Myocardial Deformation

**Fig. 5:**
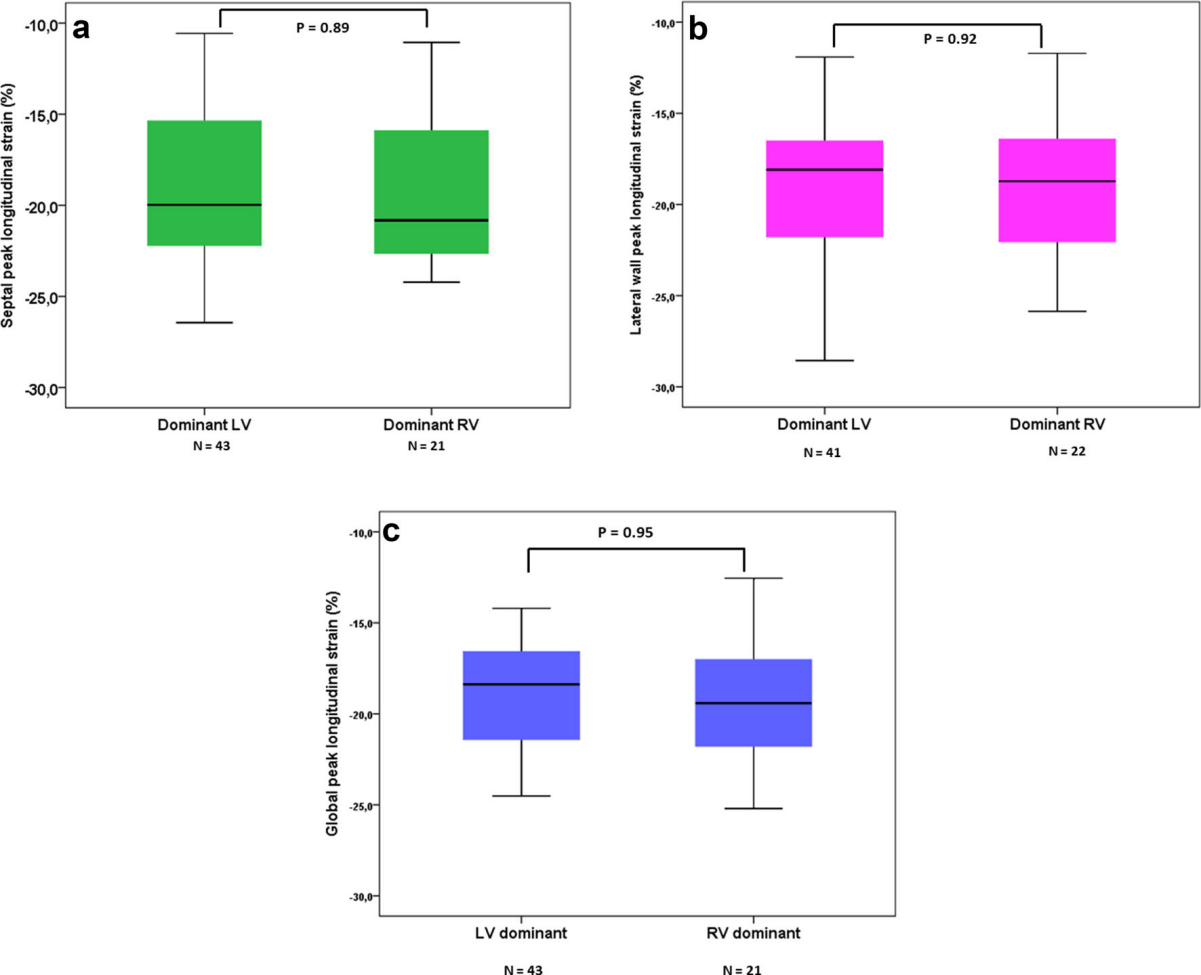
Differences between morphological left and right ventricles septal (**a**), lateral wall (**b**), and global (**c**) peak longitudinal strain

Figure 5 shows that global, lateral wall and septal longitudinal strain was similar in TCPC patients with a dominant right and left single ventricle morphology. Also, when segmental myocardial deformation was compared no differences are observed in children with left ventricular morphology and right ventricular morphology, except for longitudinal systolic strain rate of the basal lateral segment (Table 4).

**Table 4:** Segmental lateral longitudinal strain and systolic SR, depending on type of dominant ventricle (mean ± SD)

	N	Longitudinal peak strain (%)			Longitudinal systolic SR (1/s.)		
		LV	RV	*p* value	LV	RV	*p* value
Basal-septal	50	− 16.5 ± 6.5	− 14.6 ± 5.9	0.33	− 0.93 ± 0.28	− 0.95 ± 0.35	0.85
Mid-septal	63	− 18.6 ± 5.5	− 19.6 ± 4.3	0.45	− 1.02 ± 0.28	− 1.09 ± 0.30	0.39
Apical-septal	62	− 21.0 ± 5.8	− 21.9 ± 5.9	0.55	− 1.18 ± 0.41	− 1.30 ± 0.35	0.25
Apical-lateral	58	− 19.7 ± 5.6	− 19.3 ± 5.8	0.83	− 1.16 ± 0.35	− 1.12 ± 0.35	0.69
Mid-lateral	56	− 19.3 ± 4.1	− 19.7 ± 4.9	0.80	− 1.04 ± 0.28	− 1.1 ± 0.39	0.57
Basal-lateral	59	− 18.3 ± 4.7	− 18.5 ± 4.1	0.89	− 1.19 ± 0.52	− 0.94 ± 0.22	0.01

*LV* left ventricle, *RV* right ventricle, *SR* strain rate

## Discussion

This multicenter study in a relatively large cohort of children and young adults with a TCPC shows that myocardial deformation parameters assessed by STE are not related to single ventricle function assessed by cMRI and conventional echocardiography (APSE). This study also shows that single ventricular morphology in TCPC patients is unrelated to longitudinal myocardial deformation parameters.

### Myocardial Deformation and cMRI-Derived Cardiac Assessment

We found no correlation between longitudinal strain of the single ventricle assessed by echocardiography and single ventricle ejection fraction, single ventricle volume and cardiac index assessed by MRI. Khoo et al. performed a study in single RV patients before performing the bidirectional cavo-pulmonary connection (mean age 0.42 year) combining cMRI data with myocardial deformation data assessed by speckle tracking echocardiography [14]. In this small study, the authors found highly statistically significant correlation coefficients for global longitudinal deformation (strain and SR) versus cMRI-derived RV volumes and ejection fraction in the range of 0.57–0.78 [14]. It is difficult to explain why Khoo et al. found these strong correlations between cMRI-derived parameters and STE-derived myocardial deformation parameters compared to our results. A possible explanation might be that the range in ejection fraction seems large in the study by Khoo et al., ranging from 25 to 80%, while in our study ejection fraction by cMRI was generally well preserved and less widely ranged [10, 14]. When the range of values of one parameter is limited, Pearson correlation might not be the most appropriate test to investigate an association. Another possible explanation of the differences between our study and the study by Khoo et al. is that in the later study children were much younger and had less cardiac surgical procedures. Of interest is that when we dichotomized TCPC patients with relatively low and those with normal cMRI EF, no statistically significant differences in global and lateral wall longitudinal strain and systolic SR were found (Fig. 3), suggesting that at least at a relatively young age STE is not very helpful in predicting low EF assessed by cMRI.

### Myocardial Deformation in Right Versus Left Dominant Ventricles

Conceptually, a single RV may be more likely to fail over time compared to a single LV, since a RV is not intrinsically designed to sustain systemic blood pressures. Studies in patients with a systemic RV due to transposition of the great arteries (TGA, either D-TGA after Mustard/Senning operation or congenitally corrected TGA without surgical intervention) show that short- and mid-term ventricular function is generally well preserved, but that the majority of patients develop clinical RV failure after the age of 25 years [15, 16]. Many studies have investigated differences in clinical outcome, exercise capacity, and single ventricular function in patients with single RV and LV morphology with a TCPC and found conflicting results [9]. We found that STE-derived global longitudinal myocardial deformation parameters were comparable in children with LV and RV morphology, which is in agreement with a relatively large study by Petko et al. in children with a mean age of 7.8 years [9]. However, Kaneko et al. [17] found in a small study (20 children with TCPC, mean age 3.4 years) that global peak longitudinal strain was − 16.6% in children with RV morphology versus − 20.5% in children with LV morphology (*p* = 0.01). Studies performed in TCPC patients with only one type of single ventricle morphology report global longitudinal strain values in RV morphology ranging from − 12.0 to − 18.9% [7, 18–21] and in LV morphology ranging from − 14.2 to − 18.3% [18, 22, 23]. However, these studies are difficult to compare, since they vary in important factors, including age of study participants, time after completion of the TCPC, type of surgical procedure, type of echocardiographic equipment used, and underlying specific single ventricle morphology. In a recently published study by our group in the same population as described in this study, we found a higher single ventricle EF assessed by cMRI in children with LV morphology compared to children with RV morphology (55 ± 8% vs 49 ± 9%, *p* = 0.001) [10], suggesting a slightly better preserved global systolic ventricular function in TCPC patients with LV morphology. We also found indications for less favorable diastolic ventricular performance in children with RV morphology compared to LV morphology. Further studies are required to obtain more insight into this topic.

### Feasibility and Reproducibility

Longitudinal deformation measurements by STE in patients with a TCPC were feasible in 65–81% (segmental) and > 80% (global) of the participants. We have previously reported feasibility figures for normal children in the range of 91–96% for segmental longitudinal deformation and 97–100% for global longitudinal deformation [6, 24]. Previous studies using STE in children and adults with single ventricle morphology report feasibility between 63 and 100% for longitudinal strain [9, 19, 25], although some studies do not provide information on feasibility [17, 22, 26]. Based on our own experience and publications by other groups, we conclude that feasibility of STE in patients with single ventricle morphology is modest, probably due to poor acoustic window as a result of abnormal anatomy (e.g., missing segments in the setting of large ventricular septum defects) and/or various thoracotomies.

Reproducibility data are not reported in many studies using STE in children and adults with a TCPC [8, 17, 27]. Some studies use inter-class correlation coefficients to report on intra- and inter-observer reproducibility and found figures in the range of 0.8 for both longitudinal strain and SR [19, 26]. Others use COV which were found to be between 3 and 25% for both strain and SR. [9, 18, 22]. Our data on reproducibility are comparable with these studies and confirm that performing STE is challenging in patients with single ventricle morphology. Singh et al. have clearly shown that reproducibility of STE is worse in children with a TCPC compared to subjects with anatomical normal hearts [23].

### Strength and Limitations

The major strengths of this study are the relatively large number of TCPC patients included and the ability to compare a number of potentially relevant functional parameters, including extensive echocardiographic data (both conventional and innovative parameters such as strain and SR) as well as cMRI data. Other studies using myocardial deformation in single ventricle patients with a TCPC often included rather small numbers of patients [7, 8, 17–19, 21–23, 28] which might result in lack of statistical power.

A recent study by Ghelani et al. investigated the comparability of myocardial deformation assessed by echocardiography and myocardial deformation assessed by cMRI using vendor independent software [29]. They found that intermodality agreement was modest for circumferential strain and poor for longitudinal strain [29]. We decided not to use the vendor independent software, since this software package uses DICOM data with reduced frame rate, which hampers the analysis of strain rate, since this parameter is highly dependent on adequate frame rate. We however acknowledge that it would be interesting to compare myocardial strain assessed by vendor-dependent software with cMRI strain.

We decided to only include longitudinal deformation in our study. In contrast to the experience of Ghelani et al., who used circumferential strain and SR in a rather large study in young adults with a TCPC [30], we found it very difficult to measure circumferential strain in our study population. By definition, when studying a heterogeneous single ventricle population, many participants will (at least partly) miss their ventricular septum, making it very hard to study circumferential strain. We therefore believe that circumferential strain is an unlikely parameter to become of clinical use in following myocardial function in the majority of TCPC patients.

Finally, although sample size of our study is considerably larger compared to previously published studies, data were missing in some participants for echocardiographic or cMRI parameters due to poor echocardiographic acoustic window, the presence of a pacemaker device or claustrophobia.

### Clinical Implications

The present study emphasizes that echocardiography and cMRI are complementary diagnostic tests to evaluate the cardiac function of TCPC patient. Our results provide little support that myocardial deformation by STE might have a role in the assessment of this patient group, at least at this relatively young age. Since our study was of cross-sectional design, we cannot exclude that STE might have a role in predicting single ventricle failure in follow-up studies and further investigations on this topic are warranted.

## Conclusions

Myocardial function in contemporary TCPC patients with univentricular hearts is on average well preserved in children and young adults. Longitudinal myocardial deformation by STE, a novel non-invasive technique yielding relatively low costs, does not predict the presence of low EF by cMRI. Clinical utility is also hampered by moderate feasibility and variability of the current available techniques. Based on STE-derived parameters, the myocardial function in morphological RVs is similar compared to morphological LVs.
